# SOX2 boosts major tumor progression genes in prostate cancer and is a functional biomarker of lymph node metastasis

**DOI:** 10.18632/oncotarget.6029

**Published:** 2015-10-19

**Authors:** Marco Vincenzo Russo, Silvia Esposito, Maria Grazia Tupone, Lamberto Manzoli, Irma Airoldi, Paolo Pompa, Luca Cindolo, Luigi Schips, Carlo Sorrentino, Emma Di Carlo

**Affiliations:** ^1^ Department of Medicine and Sciences of Aging, “G. d'Annunzio” University, Chieti, Italy; ^2^ Ce.S.I. Biotech, Aging Research Center, “G. d'Annunzio” University Foundation, Chieti, Italy; ^3^ Laboratory of Oncology, Istituto “Giannina Gaslini”, Genova, Italy; ^4^ Operative Unit of Urology, “Santo Spirito” Hospital, Pescara, Italy; ^5^ Department of Urology, “San Pio da Pietrelcina” Hospital, Vasto, Italy

**Keywords:** prostate cancer, laser capture microdissection, neuroendocrine differentiation, SOX2, metastasization

## Abstract

Critical issues in prostate cancer (PC) are *a.* identification of molecular drivers of the highly aggressive neuroendocrine differentiation (NED) in adenocarcinoma, and *b.* early assessment of disease progression.

The SRY (sex determining region Y)-box 2 gene, *SOX2*, is an essential embryonic stem cell gene involved in prostate tumorigenesis. Here we assessed its implications in NED and progression of PC and its diagnostic and prognostic value.

Laser microdissection, qRT-PCR, quantitative Methylation-Specific PCR and immunohistochemistry were used to analyze *SOX2* gene expression and regulation in 206 PC samples. Results were examined according to the patient's clinical pathological profile and follow-ups. Functional studies were performed using PC cells transfected to overexpress or silence *SOX2*.

*SOX2* was consistently downregulated in PC, except in cell clusters lying within lymph node (LN)-positive PC. Multivariate analysis revealed that *SOX2* mRNA expression in the primary tumor was significantly associated with LN metastasis. When *SOX2* mRNA levels were ≥1.00, relative to (XpressRef) Universal Total RNA, adjusted Odds Ratio was 24.4 (95% CI: 7.54–79.0), sensitivity 0.81 (95% CI: 0.61–0.93) and specificity 0.87 (95% CI: 0.81–0.91). Patients experiencing biochemical recurrence had high median levels of *SOX2* mRNA.

In both PC and LN metastasis, SOX2 and NED marker, Chromogranin-A, were primarily co-expressed. In PC cells, NED genes were upregulated by SOX2 overexpression and downregulated by its silencing, which also abolished SNAI2/Slug dependent NED. Moreover, SOX2 upregulated neural CAMs, neurotrophins/neurotrophin receptors, pluripotency and epithelial-mesenchymal transition transcription factors, growth, angiogenic and lymphangiogenic factors, and promoted PC cell invasiveness and motility.

This study discloses novel SOX2 target genes driving NED and spread of PC and proposes SOX2 as a functional biomarker of LN metastasization for PC.

## INTRODUCTION

Prostate Cancer (PC) is a heterogeneous disease that ranges from asymptomatic to a rapidly fatal systemic malignancy with the features of small-cell neuroendocrine cancer, or neuroendocrine differentiation (NED) nests, which arise in conventional adenocarcinoma [1]. Identification of genetic drivers of these variants and molecular determinants of PC spreading should be not only of great prognostic value, but essential for a targeted prevention or therapy of metastatic disease. Recent studies have revealed that pluripotency associated genes may condition the biological heterogeneity of a cancer and correlate with its aggressiveness [2]. The SRY (sex determining region Y)-box 2 gene, *SOX2*, so far recognized as crucial for the stem cell state [3] and necessary for induced cellular reprogramming [3, 4], is gaining a renewed interest as a key regulator of self-renewal and maintenance of Cancer Stem Cells (CSCs) in a variety of tumors including PC [5–10]. In this type of cancer, SOX2 has been shown to increase cellular proliferation and survival, to stimulate epithelial-mesenchimal transition (EMT) [11, 12] and to promote castration-resistant disease [13]. We recently found that the EMT transcription factor *SNAI2/Slug* upregulates *SOX2* in PC cells and that these genes are co-expressed at the invasion front and in NED areas of high-grade PC [14]. However, the question of whether *SOX2* may drive NED remains unresolved and the way it may favor PC progression is not fully elucidated.

By means of laser capture microdissection (LCM) followed by molecular and genetic analyses, we assessed *SOX2* gene expression and regulation in PC samples and correlated the molecular data to the patient's clinical pathological profiles and follow-ups. We also performed *in vitro* studies with human PC cell lines to investigate *SOX2*'s ability to regulate critical PC progression pathways.

## RESULTS

### *SOX2* is downregulated in PC and its expression correlates with NED and lymph node metastasization

We recently found that *SOX2* was downregulated, as observed for *SNAI2*, in the neoplastic prostatic epithelia compared to its normal counterpart [14]. In the present study, we investigated the possible mechanisms involved in its regulation and extended molecular and immuno-pathological analyses to a greater number of PC samples (from a new cohort of patients) to find out whether *SOX2* expression may be related to the patient's clinical pathological characteristics and follow-ups.

**Figure 1:**
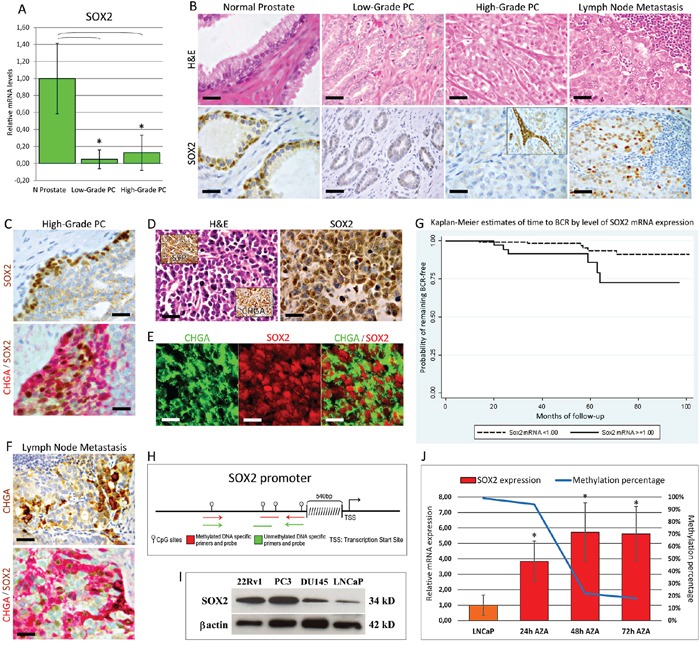
Expression of SOX2 in normal and neoplastic prostate tissue from PC patients. A. *SOX2* mRNA expression in microdissected normal and cancerous prostate tissues from PC patients Histogram representing the relative expression ± SD of *SOX2* mRNA in normal epithelium and its neoplastic counterparts with low or high Gleason grades, from clinical prostate samples of patients subjected to radical prostatectomy, normalized with the housekeeping gene *HPRT*. One-way ANOVA was used for comparisons between epithelial compartments of normal prostate and low- and high-grade PC: *p* < 0.0001. *p < 0.01 Tukey HSD Test compared with normal prostate epithelium. **B.** SOX2 protein expression in normal and cancerous prostate tissues and lymph node metastases from PC patients. Immunohistochemistry shows that normal prostate glands display a bright SOX2 nuclear expression in most of the basal epithelial cell layer. SOX2 expression was frequently lost in the malignant epithelium of PC with either low or high Gleason grades, except for some low- or more frequently high-grade PC foci showing stromal infiltrating SOX2-positive cancer cell clusters (inset). Most lymph node metastases displayed a distinct to strong nuclear expression of SOX2. Magnification: X630, top and bottom left panels; X400, the remaining panels. Scale bars: 20 μm (top and bottom left panels); 30 μm (the remaining panels). **C.** SOX2 protein expression in high-grade PC. Immunohistochemistry shows a strong SOX2 expression confined to cancer cell clusters bordering the expansion/invasion fronts, which frequently co-expressed CHGA. Magnification: X630. Scale bars: 20 μm. **D.** SOX2 protein expression in neuroendocrine (NED) areas of high–grade PC. Immunohistochemistry shows a strong SOX2 expression in the majority of CHGA+/SYP+ (insets) neoplastic cells forming NED areas. Magnification: X630. Scale bars: 20 μm. **E.** Expression of CHGA and SOX2 in NED areas. Confocal analyses of double immunofluorescence staining for CHGA (green) and SOX2 (red) show that CHGA+ cells express SOX2 in their nuclei. Magnification: X630. Scale bars: 20 μm. **F.** Expression of CHGA and SOX2 in lymph node metastasis. Lymph node metastasis frequently include scattered CHGA-positive cancer cells mostly endowed with nuclear SOX2 positivity, as shown by the double SOX2 (brown) / CHGA (red) immunostaining. Magnification: X630. Scale bars: 20 μm. **G.** Kaplan-Meier estimates of time to biochemical recurrence (BCR) by level of *SOX2* mRNA expression. **H.** DNA methylation status of *SOX2* gene promoter in microdissected PC epithelia and in LNCaP cell line. The last portion of the *SOX2* promoter, 540bp upstream from the transcription start site, contains a dense CpG 128bp sequence. **I.** WB analyses of SOX2 expression in PC cell lines. SOX2 protein expression is substantial in 22Rv1, PC3, and DU145 cell lines, whereas it is barely detected in LNCaP cells. **J.** Restoration of *SOX2* expression by 5-Aza-dC treatment and concomitant reduction of the methylation percentage of the *SOX2* gene promoter. Expression level of *SOX2* mRNA (expressed as mean ± SD) is significantly (*p < 0.05 Student's *t*-test compared with control cells) increased in LNCaP cells, starting 24 hours after the treatment with 5-Aza-dC, whereas *SOX2* methylation status simultaneously decreases. Data are representative of three independent experiments.

In the present cohort of 206 prostatectomized PC patients, the mean level of *SOX2* mRNA was found to be significantly (*p* < 0.05) downregulated in the neoplastic epithelium from both low- and high- Gleason grade PC foci (≤3 and >3), by 20.78 and 7.87 times, respectively (with no substantial differences between them), compared to the normal counterpart (whose expression levels were similar to those in the normal epithelium of the controls) (Figure 1A).

Immunohistochemistry corroborated the molecular data and demonstrated distinct SOX2 expression in the basal cell layer of normal prostate glands (as previously reported in ref. 13) and its absence in most of the neoplastic epithelia (Figure 1B), with the exception of a few low-grade foci (10/75:13%) and a discrete number of high-grade PC foci (47/131: 36%). In the latter, SOX2 was usually localized in cell clusters infiltrating the stroma (Figure 1B) or bordering the expansion/invasion fronts, which were frequently Chromogranin-A(CHGA)-positive (Figure 1C). Furthermore, the 23 CHGA/synaptophysin (SYP)-positive NED areas (detected in 131 high-grade PC foci) displayed distinct to strong SOX2 expression (Figure 1D and 1E). Fisher's exact test, revealed a significant (*p* < 0.0001) link between the expression of SOX2 and of CHGA in the NED areas of high-grade PC.

In 21/27 node-positive PC cases a distinct to strong SOX2 expression was observed in the lymph node metastases, as seen in the primary tumor. Interestingly, 17/27 lymph node metastasis displayed distinct to strong expression of both SOX2 and CHGA (Figure 1F). A significant association was also found, through Fisher's exact test, between SOX2 and CHGA expression in the lymph node metastasis (*p* = 0.04413) (Figure 1F).

Through a univariate analysis, using a *SOX2* mRNA cutoff level of 1.00 (relative to XpressRef Universal Total RNA, Qiagen), a high *SOX2* mRNA expression level was strongly associated with lymph node metastasis (*p* < 0.001; Table 1). The sensitivity was 0.81 (95% CI: 0.61–0.93) and the specificity was 0.87 (95% CI: 0.81–0.91).

**Table 1:** Clinical pathological characteristics of the samples, overall and by lymph node status (pN0 *vs* pN ≥1)

Variables	Overall (*n* = 206)	pN0 patients (*n* = 179)	pN ≥1 patients (*n* = 27)	*p*^a^
*Median follow-up in months* (IQR^b^)	47 (34)	47 (37)	52 (37)	0.7
*Age*				
Mean age in years (SD)	65.5 (6.2)	65.2 (6.0)	67.6 (7.0)	0.010
Age-class in years, %				
<64	26.7	27.9	18.5	0.3
64–68	40.8	43.6	22.2	0.035
>68	32.5	28.5	59.3	0.002
*SOX2 mRNA expression level relative to XpressRef Universal Total RNA (XUTR)*				
Median (IQR^b^)	0.16 (0.87)	0.11 (0.58)	3.20 (4.25)	<0.001
0.00, %	35.0	39.7	3.7	<0.001
0.00–0.99, %	42.7	46.9	14.8	0.002
≥1.00, %	22.3	13.4	81.5	<0.001
*Gleason grade inmicrodissected foci, %*				
>3	63.6	58.1	100.0	<0.001
*Surgical Gleason score*				
<6	21.8	25.2	0.0	0.003
6	14.6	16.8	0.0	0.021
7	37.4	37.4	37.0	0.9
8	10.7	9.5	18.5	0.16
>8	15.5	9.2	44.4	<0.001
*Peri-neural invasion, %*	85.4	83.2	100.0	0.021
*Extra-capsular invasion, %*	42.2	36.9	77.8	<0.001
*Pathologic Stage,%*				
- pT2pN0M0	54.9	63.1	0.0	—
- pT3pN0M0	32.0	36.9	0.0	—
- any pTpN ≥1M0	13.1	0.0	100.0	—
*PSA levels (ng/ml), %*				
Median (IQR^b^)	8.96 (9.90)	8.66 (9.73)	11.91 (10.70)	0.010
<10	56.8	59.2	40.7	0.070
10–20	33.0	32.4	37.0	0.6
>20	10.2	8.4	22.2	0.027
*Biochemical recurrence, %*	5.8	2.2	29.6	<0.001

a Chi-square test for categorical variables, Mann-Whitney U test for continuous ones.

b IQR: Interquartile range.

The multivariate analysis, adjusting for age, PSA levels and Gleason score, showed that the increase in *SOX2* mRNA expression was significantly associated with the presence of lymph node metastasis (*OR* = 1.83 for each 1 unit increase in *SOX2* mRNA expression; 95% CI: 1.43–2.35; Table 2). Patients with *SOX2* mRNA level ≥ 1.00 relative to XpressRef Universal Total RNA (XUTR), compared to those with *SOX2* mRNA level < 1.00, showed a very high likelihood of metastasis (adjusted OR: 24.4; 95% CI: 7.54–79.0; Table 2).

**Table 2:** Results of the multiple logistic regression analyses indicative of lymph node metastasis

Variables	Adjusted Odds Ratio (95% Confidence interval)	*p*^a^
Model 1^a^		
*Age, 1 year increase*	1.08 (0.98–1.18)	0.14
*PSA levels, 1 ng/ml increase*	0.98 (0.90–1.06)	0.6
*Gleason score, 1 point increase*	2.56 (1.35–4.85)	0.004
*SOX2 mRNA expression relative to XUTR*		
< 1.00	1 (ref. category)	—
≥ 1.00	24.4 (7.54–79.0)	<0.001
Model 2^b^		
*SOX2 mRNA expression, 1 unit increase relative to XUTR*	1.83 (1.43–2.35)	<0.001

a Logistic regression model with 206 observations; Hosmer-Lemeshow goodness of fit *p* = 0.89; Area under the ROC curve 0.92.

b As model 1, with *SOX2* mRNA level treated as a continuous variable. With the exception of Gleason grade in microdissected focus, all the variables that have not been included in the model were not significant.

All of the individuals (*n* = 12) experiencing biochemical recurrence had high median levels of *SOX2* mRNA (median ± IQR: 1.86 ± 4.08 vs 0.14 ± 0.8 relative to XUTR, *p* = 0.007). Kaplan-Meier estimates suggested a shorter time to recurrence for the individuals with higher *SOX2* mRNA expression (Figure 1G). However, the number of relapses was too scarce to allow for a meaningful multivariate analysis.

### Expression of *SOX2* in PC samples is related to its methylation status and the treatment of PC cells with 5-Aza-dC restores its expression

Since epigenetic gene silencing through DNA methylation is significant for PC progression, we tested its involvement in *SOX2* downregulation. By quantitative Methylation-Specific PCR (qMSP) we assessed, in clinical PC and in human PC cell lines, the methylation status of a dense CpG island, 128bp sequence, in the last portion of the *SOX2* promoter (Figure 1H).

We first tested *SOX2* methylation in the microdissected epithelia from a cohort of 12 patients diagnosed with low- or high-grade PC and previously examined in function of their *SOX2* mRNA expression levels. As shown in Table 3, the percentage of methylation ranged from 0.00 to 5.33% in the normal epithelia, 6.20–99.0% in low-grade PC and 0.0–100% in high-grade PC. Interestingly, 3/6 high-grade PC, with a methylation status of 0%, presented with NED areas. Spearman's rank correlation coefficient revealed an inverse correlation between *SOX2* mRNA transcript levels and the percentage of *SOX2* methylation (Spearman's ρ = −0.751; *p* < 0.0001).

**Table 3:** *SOX2* gene promoter methylation and mRNA expression levels

Samples	Gene promoter methylation (%)	Relative mRNA expression levels^a^
**Control samples**		
1	0.00	16.00
2	2.28	16.48
3	0.05	6.31
4	4.83	10.77
5	0.00	8.63
6	0.00	8.75
7	3.18	6.97
8	5.33	6.78
9	1.17	7.68
10	1.22	12.17
11	0.00	14.36
12	0.00	15.00
**Low-grade PC samples**		
1	32.47	0.00
2	6.20	0.52
3	7.10	0.00
4	44.00	0.08
5	99.00	0.00
6	13.15	0.88
**High-grade PC samples**		
7	0.00	7.13
8	100.00	0.00
9	0.00	4.12
10	0.00	8.51
11	100.00	0.38
12	27.36	2.30

a Gene expression levels are relative to XpressRef Universal Total RNA from SABiosciences.

Indeed, Western Blotting (WB) analyses showed that SOX2 expression was remarkable in 22Rv1, PC3, DU145 cells and scarce in LNCaP cells (Figure 1I), where qMSP showed 99% DNA methylation and treatment with the demethylating agent 5-Aza-dC resulted in both a consistent (*p* < 0.05) upregulation of the *SOX2* transcript level and a concomitant reduction of the methylation percentage (Figure 1J). Thus, DNA methylation is most likely involved in the downregulation of *SOX2* observed in PC.

### SOX2 regulates NED genes, neural cell adhesion molecules, and neurotrophin/neurotrophin receptor genes

Findings from clinical specimens led us to assess whether *SOX2* may regulate gene sets related to NED and tumor progression. Two cell lines, representative of androgen-dependent and -independent PC, namely 22Rv1 and PC3 cells, were transfected to overexpress *SOX2*.

**Figure 2:**
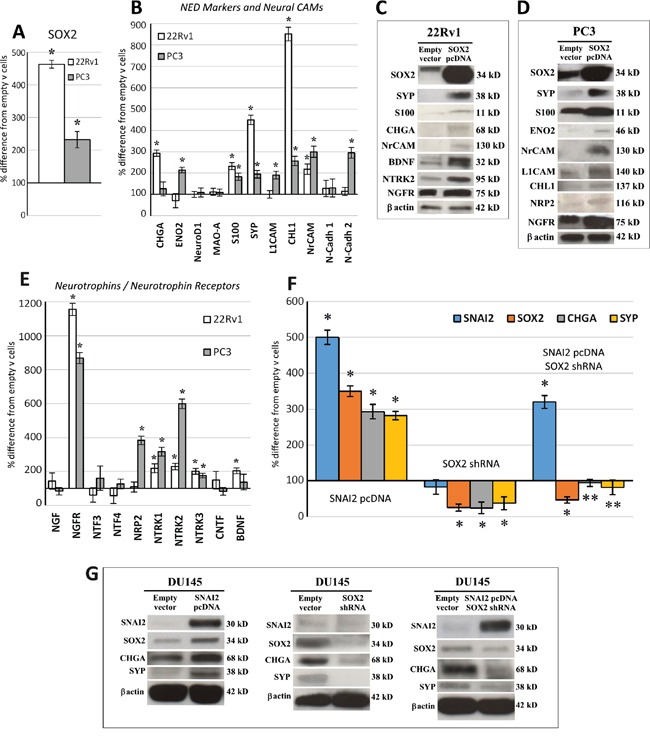
SOX2 overexpression upregulates NED, neural CAM and neurotrophin/neurotrophin receptor gene expression, and SOX2 silencing abolishes SNAI2-dependent NED **A.** qRT-PCR analyses of *SOX2* mRNA in *SOX2* gene transfected 22Rv1 and PC3 cells. Cell transfection with *SOX2* pcDNA substantially increased *SOX2* mRNA (by 363%) in 22Rv1 cells and in PC3 cells (by 132%). Data are representative of three independent experiments and results are expressed as mean ± SD. *p < 0.05 Student's *t*-test compared with control cells. **B.** Regulation of NED marker and neural CAM mRNA expression levels by *SOX2* overexpression in 22Rv1 and PC3 cell lines. *S100*, *SYP*, *CHL1* and *NrCAM* are upregulated in both *SOX2*-overexpressing cell lines, whereas *CHGA* only in 22Rv1 and *ENO2*, *L1CAM* and *N-Cadh 2* only in PC3 cells. Data are representative of three independent experiments and results are expressed as mean ± SD. *p < 0.05 Student's *t*-test compared with control cells. **C.** WB analyses of proteins extracted from empty vector and *SOX2* pcDNA transfected 22Rv1 cells. WB analysis shows *SOX2* overexpression (by 931%) in *SOX2* pcDNA transfected 22Rv1 cells and confirms, at the protein level, most of the gene expression regulation evidenced, at the transcript level, by qRT-PCR. β-actin was used as a loading control. **D.** WB analysis of proteins extracted from empty vector and *SOX2* pcDNA transfected PC3 cells. WB analysis shows *SOX2* overexpression (by 135%) in *SOX2* pcDNA transfected PC3 cells and confirms, at the protein level, most of the gene expression regulation evidenced, at the transcript level, by qRT-PCR. β-actin was used as a loading control. **E.** Regulation of neurotrophin/neurotrophin receptor mRNA expression levels by *SOX2* overexpression in 22Rv1 and PC3 cell lines. *NGFR*, *NTRK1*, *NTRK2 and NTRK3* are upregulated in both *SOX2*-overexpressing cell lines, whereas *BDNF* is upregulated only in 22Rv1 and *NRP2* only in PC3 cells. Data are representative of three independent experiments and results are expressed as mean ± SD. *p < 0.05± SD Student's *t*-test compared with control cells. **F.** Histograms represent the relative expression ± SD of *SOX2*, *SNAI2* and NED genes in the *SNAI2* pcDNA transfected, *SOX2* shRNA treated and co-transfected DU145 cells. *CHGA* and *SYP* mRNA were significantly upregulated, together with *SOX2* mRNA by *SNAI2* overexpression, whereas they were downmodulated by *SOX2* silencing. Co-transfection with both *SNAI2* pcDNA and *SOX2* shRNA abolished SNAI2-dependent *CHGA* and *SYP* upregulation. Data are representative of three independent experiments. *p < 0.05 Student's *t*-test compared with empty vector-transfected DU145 cells. **p < 0.05 Student's *t*-test compared with *SNAI2* pcDNA transfected DU145 cells. qRT-PCR data obtained from empty vector-transfected cells were similar to those obtained from untransfected cells (not shown). **G.** WB analysis of proteins extracted from *SNAI2* pcDNA transfected DU145 cells, *SOX2* shRNA treated DU145 cells and *SNAI2* pcDNA/*SOX2* shRNA co-transfected DU145 cells. WB substantiated, at protein level, the data obtained from qRT-PCR analyses showed in the panel F.

In cells transfected with *SOX2* pcDNA, the expression of both *SOX2* mRNA (22Rv1 by 363% and PC3 by 132%, *p* < 0.05) and protein (22Rv1 by 931% and PC3 by 135%, normalized to β-actin) increased substantially compared to cells transfected with the empty vector (Figure 2A).

- Within the canonical NED genes, *SOX2* overexpression upregulated *SYP* (PC3: 95%; 22Rv1: 349%) and *S100* (PC3: 83%; 22Rv1: 132%) in both cell lines, *ENO2* in PC3 cells (by 114%) and *CHGA* in 22Rv1 cells (by 193%), as assessed by qRT-PCR and WB (Figure 2B, 2C, 2D).
- Within the neural (N) Cell Adhesion Molecules (CAM), neuronal CAM (*NrCAM*), was upregulated in both *SOX2*-overexpressing cell lines (PC3: 200%; 22Rv1: 118%). Neuronal cadherin-2 (*NCadh-2*) and *L1CAM* were only upregulated in PC3 cells (by 195% and 91% respectively) with WB confirmation for the latter. Meanwhile, CAM L1-like (*CHL1)* increased in both cell lines (22Rv1: 752% and PC3: 157%), but only in PC3 cells at the protein level (Figure 2B, 2C, 2D).
- The Neurotrophin/Neurotrophin Receptor system was also influenced by SOX2. Following *SOX2* gene transfection, Brain Derived Neurotrophic Factor (*BDNF*) was upregulated in 22Rv1 cells (by 103%) and Neuropilin-2 (*NRP2*) in PC3 cells (by 285%), with confirmation at protein level. Moreover, in both cell lines, *SOX2* overexpression upregulated Neurotrophic Tyrosine Kinase Receptor type 1, *NTRK1/TrkA* (PC3: 218%; 22Rv1: 118%), and type 2, *NTRK2/TrkB* (PC3: 500%, and 22Rv1: 128%). WB analysis confirmed the result for *NTRK2/TrkB* in the 22Rv1 cells. At the transcriptional level, *NTRK3/TrkC* was induced in 22Rv1 and upregulated (by 75%) in PC3 cells, whereas Nerve Growth Factor Receptor, *NGFR/p75NTR*, was increased in PC3 (by 769%) and in 22Rv1 (by 1055%) with WB confirmation (Figure 2E, 2C, 2D).

### *SOX2* knockdown abrogates the NED gene upregulation elicited by *SNAI2/Slug* overexpression

We recently found that expression of *SNAI2/Slug*, in clinical PC samples, is associated with that of *SOX2*, and NED genes, specifically *CHGA* and *SYP*. *In vitro* experiments revealed that, in PC cells, both *SOX2* and NED genes were upregulated by *SNAI2* [14]. To assess whether *SNAI2*'s ability to boost NED genes may involve *SOX2*, we transfected DU145 cells (which have low levels of endogenous *SNAI2* mRNA with respect to other PC cell lines and low constitutive level of *SOX2* mRNA, as shown in ref. 14) to overexpress *SNAI2* and silence *SOX2*. *SOX2* silencing (by 75%) in DU145 cells resulted in a significant (*p* < 0.05) downmodulation of both *CHGA* (by 76%) and *SYP* (by 63%). Meanwhile, DU145 cells transfected with *SNAI2*-expressing vector (more than 300%, *p* < 0.05), resulted in the upregulation of *CHGA* (by 193%) and *SYP* (by 182%), as well as *SOX2* (more than 200%, *p* < 0.05) (Figure 2F) [14]. As shown in Figure 2F and 2G, knockdown of *SOX2* in *SNAI2*-overexpressing DU145 cells resulted in significantly (*p* < 0.05) lower levels of *CHGA* and *SYP* transcripts (by 199% and by 201%, respectively) and proteins, than those observed in PC cells overexpressing only *SNAI2* and comparable to the levels observed in the control vector of transfected cells.

### SOX2 regulates stemness and EMT related genes as well as growth, angiogenic and lymphangiogenic factor related genes

The significant association, emerged from the multivariate analysis, between the increase in *SOX2* mRNA expression in the primary tumor and the presence of lymph node metastasis, led us to further assess whether SOX2 regulates gene sets implicated in tumor growth and progression.

**Figure 3:**
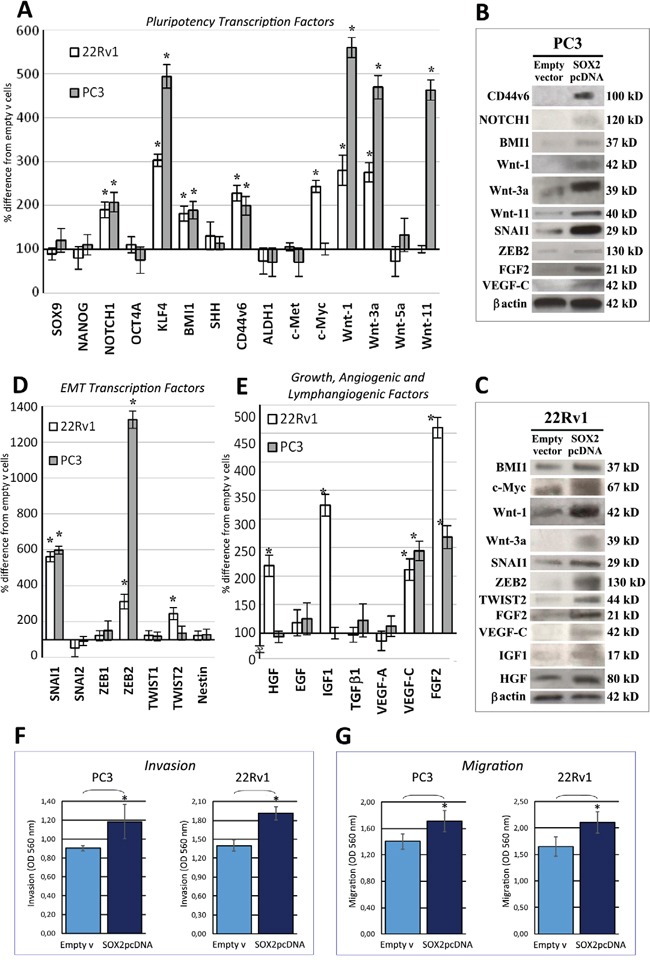
SOX2 overexpression upregulates pluripotency and EMT transcription factors, along with growth, angiogenic and lymphangiogenic factors, and promotes PC cell invasiveness **A.** Regulation of pluripotency transcription factor mRNA expression levels by *SOX2* overexpression in 22Rv1 and PC3 cell lines. *NOTCH1*, *KLF4*, *BMI1*, *CD44v6*, *Wnt-1* and *Wnt-3a* are upregulated in both *SOX2*-overexpressing cell lines, whereas *Wnt-11* only in PC3 cells, and *c-Myc* only in 22Rv1 cells. Data are representative of three independent experiments and results are expressed as mean ± SD. *p < 0.05 Student's *t*-test compared with control cells. **B.** WB analyses of proteins extracted from empty vector and *SOX2* pcDNA transfected PC3 cells WB analysis confirmed, at the protein level, most of the gene expression regulation by *SOX2* overexpression, evidenced at the transcript level, by qRT-PCR, as shown in panel A, D, and E. β-actin was used as a loading control. **C.** WB analyses of proteins extracted from empty vector and *SOX2* pcDNA transfected 22Rv1 cells WB analysis confirmed, at the protein level, most of the gene expression regulation by *SOX2* overexpression, evidenced at the transcript level, by qRT-PCR, as shown in panel A, D, and E. β-actin was used as a loading control. **D.** Regulation of EMT transcription factor mRNA expression levels by *SOX2* overexpression in 22Rv1 and PC3 cell lines. *SNAI1* and *ZEB2* are upregulated in both *SOX2*-overexpressing cell lines, whereas *TWIST2* only in 22Rv1 cells. Data are representative of three independent experiments and results are expressed as mean ± SD. *p < 0.05 Student's *t*-test compared with control cells. **E.** Regulation of growth, angiogenic and lymphangiogenic factor mRNA expression levels by *SOX2* overexpression in 22Rv1 and PC3 cell lines. *VEGF-C* and *FGF2* are upregulated in both *SOX2-overexpressing* cell lines, whereas *HGF* and *IGF1* only in 22Rv1 cells. Data are representative of three independent experiments and results are expressed as mean ± SD. *p < 0.05 Student's *t*-test compared with control cells. qRT-PCR data obtained from empty vector-transfected cells were similar to those obtained from untransfected cells (not shown). **F.** Histograms represent invasion assay of PC3 and 22Rv1 cells transfected with *SOX2* pcDNA or empty vector as measured by Optical Density (OD). Invasion assay shows a significant increase in the number of invading PC3 and 22Rv1 cells 40 hours after *SOX2* pcDNA transfection, when compared with empty vector transfected cells, as measured by OD at 560 nm after extraction, using a SpectraMax 190 microplate reader. Experiments were performed in triplicate. Results are expressed as mean ± SD. *p < 0.05 Student's *t*-test compared with control cells. **G.** Histograms represent migration assays of PC3 and 22Rv1 cells transfected with *SOX2* pcDNA or empty vector as measured by OD. Migration assay shows a significant increase in the number of invading PC3 and 22Rv1 cells 15 hours after *SOX2* pcDNA transfection, when compared with empty vector-transfected cells, as measured by OD at 560 nm after extraction, using a SpectraMax 190 microplate reader. Experiments were performed in triplicate. Results are expressed as mean ± SD. *p < 0.05 Student's *t*-test compared with control cells. Migration and invasion data obtained from empty vector-transfected cells were similar to those obtained from untransfected cells (not shown).

- *SOX2* overexpression significantly (*p* < 0.05) upregulated, in both 22Rv1 and PC3 cell lines, the expression levels of specific pluripotency genes such as *KLF4* (PC3: 394%; 22Rv1: 203%), *CD44v6* (PC3: 99%; 22Rv1: 128%), *BMI1* (PC3: 89%; 22Rv1: 81%) and *NOTCH1* (PC3: 107%; 22Rv1: 90%). WB analysis confirmed the upregulation of CD44v6 and NOTCH1 in PC3 cells and of BMI1 in both cell lines. Assessment of *c-Myc*, which has a prominent role in PC stem-like cells (PCSCs) / cancer initiating cells (CICs) maintenance and tumorigenicity [15], revealed a substantial upregulation, at the transcriptional (by 143%) and at the protein level, only in 22Rv1 cells (Figure 3A, 3B, 3C).
- Assessment of the *Wnt* family members, also involved in self-renewal, migration and survival of CSCs [16], revealed an increase (*p* < 0.05) of *Wnt-1* (PC3: 460%, 22Rv1 180%), and *Wnt-3a* transcripts (PC3: 370%; 22Rv1: 175%) and proteins in both *SOX2*-overexpressing lines and an increase of *Wnt-11* (by 363%) in PC3 cells only (Figure 3A, 3B, 3C).
- Drivers of cell invasiveness, within the EMT transcription factors, *SNAI1/Snail* and in particular, *ZEB2* transcripts and proteins, were consistently (*p* < 0.05) upregulated in both *SOX2*-overexpressing PC3 (SNAI1 by 498% and ZEB2 by 1225%) and 22Rv1 cells (SNAI1 by 462% and ZEB2 by 212%), whereas *TWIST2* was increased in the latter cell line (by 144%) (Figure 3D, 3B, 3C).
- Within the families of growth, angiogenic and lymphangiogenic factors critically involved in prostate tumorigenesis, in both *SOX2*-overexpressing cell lines, we found a considerable upregulation of *FGF2* (PC3; 168%; 22Rv1: 360%) and *VEGF-C* (PC3: 144%; 22Rv1: 111%), whereas *IGF1* and *HGF* were upregulated (by 224% and 118%, respectively) in 22Rv1 cells as confirmed by WB (Figure 3E, 3B, 3C).

### *SOX2* overexpression in PC cells promotes their invasion and migration

The correlation between *SOX2* expression by the primary tumor and the development of lymph node metastasis, together with the finding of SOX2-dependent upregulation of EMT transcription factors, neural CAMs, which mediate cell adhesion and migration [17–19] and members of the *Wnt* gene family, induced us to assess alterations in migration and invasion capabilities of *SOX2*-overexpressing PC3 and 22Rv1 cells. Both *SOX2* gene transfected lines displayed a significantly (*p* < 0.05) increased invasion (across matrigel-coated inserts) and migration ability (through a polycarbonate basement membrane) when compared to empty vector transfected cells (Figure 3F and 3G).

## DISCUSSION

A significant role is emerging for the stemness transcription factor *SOX2* in PC progression [5–6], but the dynamics of its expression and regulation in clinical samples, the related molecular outcomes and clinical pathological impact are mostly unknown. This study reveals that SOX2, regularly expressed in the basal cell layer of normal prostatic glands, is substantially downregulated, most likely by gene promoter methylation, in PC epithelia and cell lines, as previously observed in gastric cancer [20]. Although the lack of basal cells is a typical histologic feature of PC, it has emerged that this type of tumor originates in basal cells and subsequently evolves to adenocarcinoma, which is maintained by more differentiated luminal-like cells [21]. It may thus be conceivable that the epigenetic mechanisms of pluripotency gene silencing accompanying differentiation in developing embryos [22] may aberrantly occur in prostate carcinogenesis. Contrasting data on SOX2 expression in cancerous *versus* normal prostate have been reported [9, 23]. Our results from microdissected epithelia rule out confounding information from the heterogeneous stromal component and suggest that SOX2 plays a role in the normal prostatic gland hierarchy and self-renewal, whereas its epigenetic silencing accompanies the onset of most malignant glands. Its focal expression, instead, shapes NED in PC and confers selected clones with invasive and migratory properties, as corroborated by gene transfection, by functional studies and by the finding of a strong association between *SOX2* mRNA expression in the primary tumor and lymph node metastasis.

Besides small-cell/neuroendocrine PC [14, 23], other neuroendocrine tumors have been described to express SOX2, such as small-cell lung cancer and Merkel cell carcinoma of the skin [8, 24]. *SOX2* overexpression by PC cell lines, either androgen-dependent or -independent, dramatically boosts a range of NED genes such as *SYP*, *S100*, *CHGA* and *ENO2*. We recently found that SNAI2 elicits *SOX2* and NED gene expression in PC cell lines and that these genes frequently co-localized in the primary tumors [14]. The present study revealed, through co-transfection experiments, that SOX2 plays a key role in SNAI2's ability to regulate NED gene expression, since its silencing almost abolished CHGA and SYP upregulation by SNAI2. Moreover, the frequent co-expression of SOX2 and CHGA in both primary tumors (localized not only in typical NED areas, but also in PC cells scattered or located at the invasion fronts of high grade PC) and in lymph node metastasis, is highly suggestive of their cooperation in shaping the metastatic phenotype. The leading role of SOX2 in this context is also corroborated by its ability to boost a network of pro-metastatic genes such as those coding for neural tissue associated molecules *NrCAM*, *L1CAM* and *CHL1*, which behave as physiological signaling transducers of axon guidance and neuronal migration, but also as aberrant drivers for tumor cell invasion and motility [17–19].

Well-known regulator of neuronal differentiation, survival and growth, within the classic neurotrophins family, BDNF and its receptor NTRK2/TrkB, which are implicated in PC progression [25], emerge as novel SOX2 targets together with NGFR, which instead may function as a PC suppressor [26]. However, the wide range of SOX2 activated tumor progression pathways may overcome the effects of this molecular alteration.

The correlation between the expression of SOX2 in the primary tumor and lymph node metastasization fits well with its ability to upregulate critical tumor promoting factors such as FGF2 [27], VEGF-C and NRP2. Originally identified as a semaphorin receptor and regulator of axon guidance, NRP2 has been found to act as a co-receptor of VEGF-C [28], and it has been implicated in angiogenesis, lymphangiogenesis and lymph node metastasization. The first step of metastasization is invasion, which is enabled by EMT [29] and may be promoted by SOX2 through SNAI1/Snail, TWIST2 and ZEB2 upregulation, whereas migration may be driven by the IGF1/IGFR [30, 31] or HGF/Met [32, 33] signaling axis, both to be included as novel SOX2 targets. HGF has also been shown to play a role in the maintenance of PCSCs/CICs in an autocrine and paracrine fashion [34] and, together with the pluripotency genes *NOTCH1* [35], *BMI1* [36], *CD44v6* [37] and the proto-oncogene *c-Myc* [15], may be envisaged as downstream SOX2 targets which cooperate in conferring PC cells with a pluripotent and spreading-prone phenotype.

Correlated with PC aggressiveness through their ability to increase the self-renewal of putative PCSCs, promote EMT and stimulate AR target genes, members of the Wnt signaling family, namely Wnt-1, Wnt-3a and Wnt-11 [16], are substantially upregulated by SOX2 and their increased expression may lead to androgen deprivation therapy failure and PC recurrence [16].

Although Kaplan-Meir curves suggest a correlation between biochemical recurrence-free survival and *SOX2* mRNA expression, the low relapse rate observed in our cohort does not allow to draw a definitive conclusion. Even so, while disclosing a discrete network of novel SOX2 target genes mostly involved in PC progression, the novelty of our study consists in I. the revelation of a variety of molecular alterations elicited by SOX2 overexpression, which can lead to disease aggressiveness and progression, II. the identification of SOX2 as a critical target through which to hinder metastasization, and finally III. the proposal to assess SOX2 expression in the prostate needle biopsy as a biomarker of nodal metastasization and a useful tool for tailoring the extent of lymphadenectomy at surgery. A larger cohort of patients and clinical samples will be used to determine whether assessment of *SOX2* mRNA in PC might improve accuracy of current nomogram predicting LN invasion [38] and thus enter in clinical practice.

## MATERIALS AND METHODS

### Ethics statement

Written informed consent was obtained. Investigation has been conducted in accordance with the ethical standards with the Declaration of Helsinki and national and international guidelines and has been approved by the Ethical Committee of the “G. d'Annunzio” University of Chieti and Local Health Authority No.2 Lanciano-Vasto-Chieti, Italy (PROT 1945/09 COET of 14/07/2009).

### Patients and samples

We collected prostate specimens and clinical pathological data related to 206 patients who underwent radical prostatectomy for PC between 2006 and 2014 at the “S.S. Annunziata” Hospital, Chieti, Italy (Table 1), which were followed-up for 1–9 years after prostatectomy. In addition, we obtained normal prostates (histologically negative for PC or benign prostatic hyperplasia) from 15 untreated patients aged 55 to 64, prostatectomized for bladder cancer (controls). Details on the clinical pathological data and tissue sample processing are reported in the Supplementary Materials and Methods.

### Immunohistochemistry

Immunohistochemistry and immunofluorescence stainings were performed as reported in ref. 39 and described in detail in the Supplementary Materials and Methods.

### Morphometric analyses

SOX2, CHGA and SYP expression in primary tumors or lymph node metastases was evaluated, as previously reported [14], using the following criteria:

1. *the widening of the staining* expressed as the percentage of tumor or lymph node metastasis stained i.e.: <50%, ≥50% ≤70% and >70%,
2. *the strength of the staining* defined as negative (0), slight (1), moderate (2) or intense (3).

Thus, immunostaining was defined as:

• ***strong*** ++, when a) the widening was >70% and its strength ranged from slight (1) to intense (3), or b) the widening was >50% ≤70% and its strength ranged from moderate (2) to intense (3);
• ***distinct*** +, when a) the widening was >50% ≤70% and its strength was slight (1) or b) the widening was = 50% and its strength ranged from slight (1) to intense (3);
• ***absent*** −, when the widening was <50% and its strength was slight (1) to negative (0).

Immunostained formalin fixed and paraffin embedded sections were evaluated with Leica DM 2500 microscope at X400 magnification in a 85431.59 μm^2^ field, by two pathologists (EDC and CS) in blind fashion, with very good agreement (κ value = 0.82) [40].

### LCM

For LCM, 10 μm frozen sections from cancer and normal prostate specimens from the controls and PC patients were analyzed. Neoplastic foci with low Gleason grade (≤3) were microdissected from PC samples of 75/206 patients, whereas those with high-grade (>3) were obtained from the other 131 patients, as described in the Supplementary Materials and Methods.

### qMSP

For qMSP, genomic DNA was extracted from PC cell lines and from microdissected cells and bisulfite converted using the EpiTect Fast DNA Bisulfite Kit (Qiagen, Hilden, D). The methylation levels of the *SOX2* gene promoter were assessed as described in the Supplementary Materials and Methods.

### Cell cultures

PC cell lines 22Rv1, DU145, LNCaP, and PC3 were from ATCC (Manassas, VA, USA), which performed their characterization by Short Tandem Repeats profile analysis. Cell lines were passaged in our laboratory for fewer than 6 months after resuscitation. Details on cell culture, treatment with 5-Aza-2′-deoxycytidine (5-Aza-dC) and cell transfections are provided in the Supplementary Materials and Methods.

### Migration and invasion assay

The CytoSelect Cell Migration and Invasion Assay Kit (Cell-Biolabs, San Diego, CA, USA) was used as illustrated in the Supplementary Materials and Methods.

### Quantitative RT-PCR (qRT-PCR)

qRT-PCR was performed on RNA extracted from microdissected cells, PC cell lines transfected with *SOX2* pcDNA, *SNAI2* pcDNA, *SOX2* shRNA, co-transfected with *SNAI2* pcDNA/*SOX2* shRNA and respective controls, with the Quantitect Reverse Transcription Kit for the Reverse Transcription and the Quantifast SYBR Green PCR Kit for qPCR (Qiagen). Details are described in the Supplementary Materials and Methods.

### Western blotting

WB was performed using total proteins obtained from approximately 2.0 × 10^6^ cells as described in the Supplementary Materials and Methods.

### Statistical analysis

Between-group differences in the relative expression of *SOX2* mRNA in the epithelial compartments of normal and neoplastic prostate tissues were assessed by ANOVA. The difference between each pair of means was evaluated with the Tukey HSD test. Differences between *SOX2* pcDNA and empty vector transfected PC cells, in gene expression determined by qRT-PCR, in invasion and migration capability and between PC cells treated with or without 5-Aza-dC were assessed by Student's *t*-test. Differences between *SNAI2* pcDNA transfected, *SOX2* shRNA treated, *SNAI2* pcDNA/*SOX2* shRNA co-transfected, and empty vector transfected PC cells, in gene expression determined by qRT-PCR, were assessed by Student's *t*-test. Spearman's test was used to examine the correlation between *SOX2* expression levels and gene methylation status. Association between SOX2 and CHGA staining was analysed by Fisher's exact test. The potential association between the presence of lymph node metastasis and all recorded variables was initially evaluated using chi-squared test for categorical variables and the Mann-Whitney U test for continuous ones. Kaplan-Meier survival analysis was also used to examine the association between *SOX2* mRNA expression and biochemical recurrence. Stepwise forward logistic regression was then used to investigate potential independent indicators of the presence of lymph node metastasis.

Gleason grade in microdissected focus, could not be included as a variable in the logistic model, because of perfect failure prediction (100.0% of the subjects with lymph node metastasis had Gleason level higher than 3). Only significant covariates were kept into the final model and the overall number of covariates was limited to 5 throughout the steps of model fitting to reduce potential overfitting. Standard diagnostic procedures were adopted to check final model validity: influential observation analysis (Dbeta, change in Pearson chi-square), multicollinearity, interaction terms, Hosmer-Lemeshow test for the goodness of fit and C statistic (area under the Receiving Operator Curve). We had no missing values.

The *SOX2* mRNA expression level was first analyzed in each microdissected PC samples from low and high grade foci relative to XpressRef Universal Total RNA (SABiosciences, Frederick, MD, USA). We then identified a *SOX2* mRNA expression threshold among 10 arbitrarily chosen cutoffs (thresholds were identified using multiples of 0.2, starting from 0.2 up to 2.00). The criteria for the selection of the best cutoff were: higher sensitivity and specificity, > 0.70, higher Pseudo-R2 value in the logistic regression model and easiness of interpretation in clinical practice. The *SOX2* mRNA expression level of 1.00, relative to XpressRef Universal Total RNA, showed the best combination of sensitivity, specificity, and pseudo-R2 values and was, thus, reported and included in the final logistic model.

A second model, identical to the final one, was also fitted to show the results of *SOX2* mRNA expression when treated as a continuous variable (in its identifying form). All other tested cutoffs, starting from a *SOX2* mRNA expression level of 0.2, were significantly associated with lymph node metastasis in both univariate and multivariate analysis, but the results for each are not shown to avoid redundancy. Ninety-five percent confidence intervals (CI) for specificity and sensitivity were computed according to the efficient-score method (corrected for continuity) as described by Newcombe [41]. Statistical significance was defined as a two-sided *p*-value < 0.05 and all analyses were carried out using Stata version 13 (Stata Corp., College Station, TX, USA).

## SUPPLEMENTARY MATERIALS AND METHODS


