# Supplementary material for: SOX2 boosts major tumor progression genes in prostate cancer and is a functional biomarker of lymph node metastasis
Source: Oncotarget. 2015 Oct 19;7(11):12372–85. doi: 10.18632/oncotarget.6029 (PMC4914291; doi:10.18632/oncotarget.6029)
Supplement: Supplementary file 1 [file oncotarget-07-12372-s001.pdf]

SUPPLEMENTARY MATERIALS AND METHODS

Patients and samples

Patients entered in the study had not received hormone or immunosuppressive treatments or radiotherapy and were free from immune system diseases. Clinical and pathological stages were determined according to the 7th edition of the TNM Classification of Malignant Tumours [1]. Tumor grade, from the prostate biopsies, was assessed according to the Gleason scoring system [2]. The PSA values were divided into 3 classes: 1: less than 10 ng/ml; 2: 10 to 20 ng/ml; 3: more than 20 ng/ml. The biological samples were cancer and normal prostate specimens (formalin-fixed and frozen) and draining lymph nodes (formalin-fixed) from each PCa patient who underwent radical prostatectomy. Half of each normal or neoplastic tissue sample was fixed in 4% formalin and embedded in paraffin. The other half was embedded in Killik frozen section medium (Bio-Optica, Milano, Italy), snap frozen in liquid nitrogen and preserved at −80°C. For histology, paraffin embedded samples were sectioned at 4 µm and stained with H&E. Single and double immunohistochemistry was done on paraffin-embedded or frozen sections, depending on the antibody (Ab) used.

Immunohistochemistry

Immunohistochemistry on formalin-fixed, paraffin-embedded samples was performed using the primary Antibodies SOX2 (57CT23.3.4, Abcam, Cambridge, UK), CHGA (DAK-A3, Dako, Glostrup, DK) and SYP (Leica Biosystems, Newcastle Upon Tyne, UK) in a fully automated Leica Bond-Max instrument (Leica Biosystems). The immune complexes were detected using the Bond Polymer Refine Detection Kit (Leica Biosystems) according to the manufacturer's protocol. Negative controls were performed by replacing the primary Ab with 10% non-immune serum. Further controls were performed by omitting the secondary Ab. Controls were always negative.

Immunofluorescences and confocal microscopy

For double immunofluorescent staining on formalin-fixed, paraffin-embedded samples, after deparaffination, the sections were subjected to antigen retrieval in ethylenediaminetetracetic acid (EDTA) buffer at pH 9. The slides were then incubated with the first primary antibody, SOX2 (Abcam), for 30 minutes, followed by incubation with the biotinylated secondary antiboby (Vector Laboratories, Burlingame, CA, USA) and subsequently with the Alexa Fluor 594 Streptavidin conjugated antibody (Molecular Probes, Eugene, OR, USA). Next, the sections were incubated with the second primary Ab, CHGA (Dako), for 30 minutes, followed by incubation with the biotinylated secondary Ab and subsequently with Alexa Fluor 488 Streptavidin conjugated antibody (Molecular Probes). Cross-reaction between the first secondary Ab and Alexa Fluor 488 Streptavidin conjugated was prevented by saturating all of its binding sites with the Alexa Fluor 594 Streptavidin conjugated antibody. Slides were mounted with vectashield medium (Vector Laboratories) and examined with a Zeiss LSM 510 Meta laser scanning confocal microscope (Zeiss, Oberkochen, D).

Laser capture microdissection (LCM)

LCM was performed using the P.A.L.M. Micro Beam System (P.A.L.M. Microlaser Technologies, Bernried, D). For LCM, 10 µm frozen sections (two sections per sample) from cancer and normal prostate specimens (of both control and PCa patients) were mounted on polyethylene naphthalate membrane-covered slides (P.A.L.M. Microlaser Technologies), thawed at room temperature, and immersed in cold acetone (5 min). Immediately after H&E staining, the sections were used for LCM. From 1000 to 1500 selected cells were cut and catapulted intact into the cap of an LPC-Microfuge Tube (P.A.L.M.), and RNA was immediately isolated with the RNeasy Plus Micro Kit (Qiagen, Hilden, D). Two sections per sample were analyzed. Epithelial components were dissected from cancer and normal prostate specimens (of both control and PCa patients) discriminating between neoplastic foci with low (well differentiated) *versus* high (poorly differentiated) Gleason grade (≤3 *versus* >3). Since the Gleason score is the sum of the grade assigned to the most common tumor pattern and the grade assigned to the next most common tumor pattern, to analyze homogeneous cell populations, only cells of the tumor foci with the highest Gleason grade were microdissected from each patient's tissue specimen. Foci with the same grade were microdissected from each specimen. Foci with low Gleason grade (≤3) were microdissected from PC samples of 75/206 patients, whereas those with high-grade (>3) were obtained from the other 131 patients.

All reagents used for LCM were prepared with Ultrapure DNase/RNase-free distilled water (Invitrogen, Paisley, UK).

Methylation analysis by quantitative Methylation-Specific PCR (qMSP)

The methylation levels of the *SOX2* gene promoter, was performed by qMSP with primers and TaqMan probes specific for the methylated and the unmethylated

converted DNA, for amplification and detection of a 128bp sequence located in the last portion of the promoter, 540bp upstream from the transcription start site. To detect insufficient bisulfite conversion, primers and probe for the housekeeping gene *HPRT*, specific for the unconverted DNA, were used. All primers and probes were designed with Beacon Designer software (Premier Biosoft, Palo Alto, CA, USA) in our laboratory and synthesized by Sigma-Aldrich Corporation (Sigma-Aldrich, St.Louis, MO, USA): Methylated *SOX2* forward (5′-ATAGAGTAAGTTACGTGGAAGTAAGGAAGG-3′); Methylated *SOX2* reverse (5′-CGCTCTCCTCTCTTAC CTTACAAATACC-3′); Methylated *SOX2* probe (5′-[6FAM]CTCTCACGCCCTTCTCACAATCCCGACTAA [BHQ1]-3′); Unmethylated *SOX2* forward (5′-ATAGAGTAAGTTATGTGGAAGTAAGGAAGG-3′); Unmethylated *SOX2* reverse (5′-TCCACTCTCCT CTCTTACCTTACAAAT-3′); Unmethylated *SOX2* probe (5′-[6FAM]CAACACTCTCTCACACCCTTCT CACAATCC[BHQ1]-3′); Unconverted *HPRT* forward (5′-GGGAGAGATTCATTTATTTTAAG-3′); Unconverted *HPRT* reverse (5′-GGCCTTAATAGAACAAACA-3′); Unconverted *HPRT* probe (5′-[6FAM] AAGGCATTCTCC AGCACATTG[BHQ1]-3′). qMSP was performed using the QuantiFast Multiplex PCR Kit (Qiagen) on the MJ Mini Opticon Real-Time PCR System (Bio-Rad, Hercules, CA, USA) under the following conditions: 95°C for 5 min; 95°C for 30 sec; 67°C for 30 sec, repeated for 44 cycles. The relative percentage of methylated DNA was determined using the formula: $Cmeth = 100/(1+2^{Ct\ CG-CtTG})$%.

Treatments with 5-Aza-2′-deoxycytidine and cell transfection with *SOX2* expressing vector

PC cell lines 22Rv1, DU145, LNCaP, and PC3 (from ATCC, Manassas, VA, USA) were cultured in RPMI-1640 with 10% FCS.

LNCaP cells were cultured in the presence or absence of 5-Aza-2′-deoxycytidine (5-Aza-dC) (AZA, 5-10 μM, Sigma-Aldrich) for 24-72 hours. Every 24 hours, RNA was extracted using RNeasy Mini Kit (Qiagen) and subjected to real time RT-PCR using the Quantifast SYBR Green PCR Kit (Qiagen).

For overexpression of *SOX2* in PC3 and 22Rv1 cells, the GFP-tagged *SOX2* expression vector and the empty vector pCMV6-AC-GFP (both from OriGene Technologies, Rockville, MD, USA) were transfected into 1×10^6 cells using lipofectamine RNAiMax reagent (Life Technologies, Waltham, MA, USA).

For silencing of *SOX2* in DU-145 cells, two SureSilencing shRNA plasmids for human *SOX2* (GFP-tagged) and the correspondent negative control shRNA plasmid (Qiagen) were used. Each plasmid was transfected into 1×10^6 DU-145 cells using Lipofectamine RNAiMax reagent (Life technologies). In the co-trasfection experiments, each SureSilencing shRNA plasmid was transfected with the overexpression plasmid *SNAI2* (GFP-tagged) into 1×10^6 DU-145 cells, using lipofectamine RNAiMax reagent (Life Technologies). As negative controls, the negative control shRNA plasmid (Qiagen) and the empty vector (pCMV6- AC-GFP) (OriGene) were used.

Twenty-four, 48, and 72 hours after cell transfection, RNA was extracted and subjected to real time RT-PCR and the cell pellets were collected for Western Blotting.

Migration and invasion assay

CytoSelect™ Cell Migration and Invasion Assay Kit (Cell Biolabs, San Diego, CA, USA) was used for migration and invasion assays, according to the manufacturer′s protocol. Briefly, 24 hours after transfection, cells were counted and placed on polycarbonate membrane or matrigel inserts at 1×10^6 cells/mL in serum-free medium and were incubated for 24 hours (migration assay) or 48 hours (invasion assay) at 37°C. Cells were removed from the top of the inserts and cells that migrated and invaded through the inserts were fixed, stained, and quantified by optical density at 560 nm after extraction, using a SpectraMax 190 microplate reader (Molecular Devices, CA, USA).

Quantitative RT-PCR (qRT-PCR)

The primers for *CD44v6*, *NANOG*, *Nestin*, *OCT4A*, *SNAI1*, *SNAI2*, *SOX2*, *VEGF-A*, *TWIST1*, *TWIST2*, *ZEB1*, *ZEB2* and the housekeeping gene *HPRT* were designed and synthesized by Sigma-Aldrich Corporation: *CD44v6* forward 5′-AGGAACAGTGGTTTGGCAAC-3′ and *CD44v6* reverse 5′-CGAATGGGAGTCTTCTCTGG-3′; *SNAI1* forward 5′-CCTCTTCCTCTCCATACCT-3′ and *SNAI1* reverse 5′-TTCATCAAAGTCCTGTGGG-3′; *SNAI2* forward 5′-TGTCATACCACAACCAGAGA-3′ and *SNAI2* reverse 5′-CTTGGAGGAGGTGTCAGAT-3′; *SOX2* forward 5′-AGAGAGAAAGAAAGGGAGAGA-3′ and *SOX2* reverse 5′-AATCAGGCGAAGAATAATTTGG-3′; *TWIST1* forward5′-CGGAGACCTAGATGTCATT-3′ and *TWIST1* reverse 5′-CTGTCTCGCTTTCTCTTTT-3′; *TWIST2* forward 5′-AACTGGACCAAGGCTCTC-3′ and *TWIST2* reverse 5′-GCGGCGTGAAAGTAAGAAT-3′; *VEGF-A* forward 5′-AGGAGCCTCCCTCAGGGTTTC-3′ and *VEGF-A* reverse 5′-AGGACTGTTCTGTC GATGGTGATG-3′; *ZEB1* forward 5′- CCAACAGACCA GACAGTG-3′ and *ZEB1* reverse 5′-TGACTCGCA TTCATCATCTT-3′; *ZEB2* forward 5′-CGGAGACT TCAAGGTATAATCTATC-3′ and *ZEB2* reverse

5′-GTTACGCCTCTTCTAATGACAT-3′; *HPRT* forward 5′-AGACTTTGCTTTCCTTGGTCAGG-3′ and *HPRT* reverse 5′-GTCTGGCTTATATCCAACACTTCG-3′.

Primers for *ALDH1* (product number QT00013286), *BDNF* (product number QT00235368), *BMI1* (product number QT00052654), *CHGA* (product number QT00025018), *c-Met* (product number QT00023408), *c-Myc* (product number QT00035406), *CHL1* (product number QT00024801), *CNTF* (product number QT00092904), *EGF* (product number QT00051646), *ENO2* (product number QT00084889), *FGF2* (product number QT00047579), *HGF* (product number QT01758988), *IGF1* (product number QT00029785), *KLF4* (product number QT00061033), *L1CAM* (product number QT00094682), *MAO-A* (product number QT00040411), *N-Cadh1* (product number QT00063196), *N-Cadh 2* (product number QT00018963), *NeuroD1* (product number QT00203189), *NGF* (product number QT00001589), *NGFR* (product number QT00056756), *NOTCH1* (product number QT01005109), *NrCAM* (product number QT00006020), *NRP2* (product number QT01011794), *NTF3* (product number QT00204218), *NTF4* (product number QT00210924), *NTRK1* (product number QT00054110), *NTRK2* (product number QT00082033), *NTRK3* (product number QT00052906), *S100* (product number QT00208264), *SHH* (product number QT01156799), *SOX9* (product number QT00001498), *SYP* (product number QT00013062), *TGFβ1* (product number QT00000728), *VEGF-C* (product number QT00061579), *WNT-1* (product number QT00032053), *WNT-3a* (product number QT00220542), *WNT-5a* (product number QT00025109), *WNT-11* (product number QT00018270) were purchased from Qiagen. The qRT-PCR was done using the MJ Mini Opticon Real-Time PCR System (Bio-Rad) with SYBR Green fluorophore under the following conditions: denaturation at 95°C for 5 min. followed by 40 amplification cycles (denaturation at 95°C for 10 seconds and annealing/ extension at 60°C for 30 seconds). Melting curve analysis was done to assess the specificity of PCR products (samples were heated from 65°C to 95°C at a ramp rate of 0.3°C every 5 seconds). The efficiency of reaction for each target was evaluated by amplifying serial dilutions of cDNA. Relative quantification of mRNA was done according to the comparative threshold cycle method with *HPRT* as calibrator, using the Bio-Rad CFX Manager software. Gene expression levels are relative to XpressRef Universal Total RNA from SABiosciences (Frederick, MD, USA). The samples were processed in triplicate and wells without added cDNA served as negative controls.

Western blotting (WB)

For WB, cells were collected following standard procedures. Then, 1.0 mL of ice cold RIPA Lysis buffer (Thermo Scientific, Waltham, MA, USA) was added (supplemented with freshly added Protease and Phosphatase Inhibitors Cocktails (Thermo Scientific). Total proteins were measured in the extract by the Bradford assay. The whole cell lysates were examined on Mini-PROTEAN TGX Gels 4–20% (Bio-Rad). Proteins were transferred from the gels on Immun-Blot PVDF Membranes (Bio-Rad) in the transfer buffer (glycine, tris [pH 8.4] and methanol) using Mini Trans-Blot Cell apparatus (Bio-Rad). Membranes containing the proteins were blocked with milk 5% (Sigma-Aldrich) in TBST and subsequently, probed with primary and horseradish peroxidase conjugated secondary antibodies following standard procedures. Proteins transferred membranes were then washed with TBST and developed with Pierce ECL Western Blotting Substrate (Thermo Scientific).

The following primary anti-human antibodies were used: anti-SOX2 (clone 57CT23.3.4), anti-BMI1 (clone 1.T.21), anti-KLF4 [clone EPR3550(2)(ABC)], anti-TWIST2 (clone ab57997), anti-Wnt-3a (clone 3A6), anti-CHL1, anti-NTRK2 and anti-NTRK3 (all from Abcam); anti-Neuron-Specific Enolase/ENO2 (clone BBS/NC/VI-H14) (from Dako); anti-CD44v6 (clone VFF-7), anti-Chromogranin-A/CHGA (clone E-5), anti-c-Myc (clone 9E10), anti-IGF1 (clone W18), anti-neuropilin-2/NRP2 (clone C-9), anti-BDNF, anti-FGF2, anti-NGFR, anti-NOTCH1, anti-NTRK1, and anti-Wnt-1 (all from Santa Cruz Biotechnology, Dallas, TX, USA); anti-beta-actin (clone AC-15), anti-CDH12/N-Cadh 2, anti-HGF and anti-NrCAM (all from Sigma-Aldrich); anti-L1CAM (clone 2A6), anti-ZEB2 (clone 1E12), anti-SNAI1, anti-SNAI2/Slug (clone 1A6) and anti-Wnt-11 (all from OriGene Technologies); anti-S100 (clone 4E7E10) (from Thermo Scientific); anti-VEGF-C (from Life Technologies); anti-Synaptophysin/SYP (clone 2/Synaptophysin) (BD Biosciences, Franklin Lakes, NJ, USA).

The following secondary antibodies were used: rabbit anti-mouse IgG (whole molecule)-Peroxidase and rabbit anti-goat IgG (whole molecule)-Peroxidase (both from Sigma-Aldrich); goat anti-rabbit IgG (H + L)-HRP Conjugate (Bio-Rad).

REFERENCES

1. Sobin LH, Gospodarowicz MK, Wittekind C. TNM Classificazione dei tumori maligni, 7th ed. Milano: Raffaello Cortina Editore; 2009. p. 241–5.
2. Helpap B, Egevad L. The significance of modified Gleason grading of prostatic carcinoma in biopsy and radical prostatectomy specimens. Virchows Arch. 2006; 449: 622–627.
